# RNA Polymerase III Output Is Functionally Linked to tRNA Dimethyl-G26 Modification

**DOI:** 10.1371/journal.pgen.1005671

**Published:** 2015-12-31

**Authors:** Aneeshkumar G. Arimbasseri, Nathan H. Blewett, James R. Iben, Tek N. Lamichhane, Vera Cherkasova, Markus Hafner, Richard J. Maraia

**Affiliations:** 1 Intramural Research Program, Eunice Kennedy Shriver National Institute of Child Health and Human Development, National Institutes of Health, Bethesda, Maryland, United States of America; 2 National Institute of Arthritis and Musculoskeletal and Skin Diseases, National Institutes of Health, Bethesda, Maryland, United States of America; 3 Commissioned Corps, U. S. Public Health Service, Washington, DC, United States of America; Ohio State University, UNITED STATES

## Abstract

Control of the differential abundance or activity of tRNAs can be important determinants of gene regulation. RNA polymerase (RNAP) III synthesizes all tRNAs in eukaryotes and it derepression is associated with cancer. Maf1 is a conserved general repressor of RNAP III under the control of the target of rapamycin (TOR) that acts to integrate transcriptional output and protein synthetic demand toward metabolic economy. Studies in budding yeast have indicated that the global tRNA gene activation that occurs with derepression of RNAP III via *maf1-*deletion is accompanied by a paradoxical loss of tRNA-mediated nonsense suppressor activity, manifested as an antisuppression phenotype, by an unknown mechanism. We show that *maf1*-antisuppression also occurs in the fission yeast *S*. *pombe* amidst general activation of RNAP III. We used tRNA-HydroSeq to document that little changes occurred in the relative levels of different tRNAs in *maf1Δ* cells. By contrast, the efficiency of *N2*,*N2*-dimethyl G26 (m^**2**^_**2**_G26) modification on certain tRNAs was decreased in response to *maf1*-deletion and associated with antisuppression, and was validated by other methods. Over-expression of Trm1, which produces m^**2**^_**2**_G26, reversed *maf1-*antisuppression. A model that emerges is that competition by increased tRNA levels in *maf1Δ* cells leads to m^**2**^_**2**_G26 hypomodification due to limiting Trm1, reducing the activity of suppressor-tRNASerUCA and accounting for antisuppression. Consistent with this, we show that RNAP III mutations associated with hypomyelinating leukodystrophy decrease tRNA transcription, increase m^**2**^_**2**_G26 efficiency and reverse antisuppression. Extending this more broadly, we show that a decrease in tRNA synthesis by treatment with rapamycin leads to increased m^**2**^_**2**_G26 modification and that this response is conserved among highly divergent yeasts and human cells.

## Author Summary

Transfer RNAs (tRNAs) are molecular adapters necessary for translation of the genetic code from DNA to messenger RNA (mRNA) to synthesis of the proteins that constitute the energy producing enzymes and structural components of all cells and organisms on earth. In eukaryotic cells, tRNAs are synthesized by RNA polymerase III (RNAP III). Proteins are composed of different amino acids, sequentially arranged according to the triplet sequence code, each carried to the ribosome by a different tRNA which reads or decodes the triplet sequence of the mRNA. After their synthesis by RNAP III, tRNAs are chemically modified by enzymes on multiple of their nucleosides. Here we report that one of these modifications, dimethyl-guanine-26 (m^**2**^_**2**_G26) varies in the efficiency by which it is added to its target tRNAs, in a manner that is dependent on the overall activity rate of RNAP III. We show that this is important because the m^**2**^_**2**_G26 modification activates the tRNA for function to translate the code. This link between RNAP III activity rate and m^**2**^_**2**_G26 modification efficiency was previously unknown, and we show that it is conserved from yeast to human cells.

## Introduction

Apart from their role in translation, tRNAs can regulate gene expression [1,2] and serve as metabolic sensors [3], and their over-expression is associated with cell proliferation and transformation [4,5]. RNAP III is activated by oncogenes [6,7] whereas its repression reduces transformation and tumorigenesis [8].

Accumulating evidence indicate the importance of matching tRNA activity with mRNA codon demand [9]. Different cells and tissues show differences in tRNA abundances that vary with codon use [1,10]. tRNA specific activity for codon-specific decoding can be controlled by posttranscriptional modifications, most notably in the anticodon loop for nucleosides at wobble position 34 and position 37 [2,11–14].

Despite RNAP III ubiquity in eukaryotic cells, mutation in one of its catalytic subunits can manifest as a tissue-specific developmental defect in zebra fish [15,16]. In humans, mutations in either of the two catalytic subunits lead to a nervous system disorder, hypomyelinating leukodystrophy (HLD) and other tissue-specific defects ([17] and refs therein), although how these mutations affect RNAP III transcription and cause disease is unknown.

The highly conserved RNAP III repressor, Maf1, acts in response to stress including lack of nutrient, and in *S*. *cerevisiae*, mammals and other species, is under the control of the target of rapamycin (TOR) kinase [18,19], which integrates information from several environmental cues and stress states, and functions to sustain growth and homeostasis in various conditions [20]. When Maf1 is nonfunctional, cells produce much increased and unregulated transcription by RNAP III, the energy cost of which is wasted, highlighting a function for Maf1 as a key contributor to metabolic economy [21].

A striking phenotype of *S*. *cerevisiae maf1-*mutants is antisuppression [19,22] which reflects loss of suppressor-tRNA (sup-tRNA)TyrUUA mediated suppression of a nonsense codon in a mRNA encoding an adenine metabolic enzyme. Although described nearly 20 years ago and to date only for *S*. *cerevisiae*, *maf1-*antisuppression is paradoxical because it occurs amidst global increases in tRNA synthesis [19,22,23].

We deleted *maf1*+ from *S*. *pombe* and also observed antisuppression, in this case by sup-tRNASerUCA, amidst general increases in tRNA levels. We employed a tRNA-enriched limited hydrolysis sequencing method, termed tRNA-HydroSeq, on *S*. *pombe maf1*+, *maf1Δ* and other strains. While the levels of different tRNAs relative to each other varied little upon *maf1*+ deletion or over-expression, consistent with global regulation, a sup-tRNASerUCA modification, N2,N2-dimethylguanosine-26 (m^**2**^_**2**_G26), was specifically decreased in *maf1Δ* and shown to be required for efficient suppression. Trm1 is a nuclear enzyme that produces m^**2**^_**2**_G26 which likely contributes to proper tRNA folding [24,25] (see Discussion). Trm1 activity is limiting in the context of increased tRNA production in *maf1Δ* cells and we show that its over-expression reverses antisuppression. Treatment with rapamycin or over-expression of *maf1*+ reduces tRNA transcription with increase in the m^**2**^_**2**_G26 content of sup-tRNASerUCA and its specific activity for suppression. We also introduced mutations in a catalytic subunit of RNAP III associated with hypomyelinating leukodystrophy (HLD) to show that a general decrease in tRNA transcription by another mechanism also increases m^**2**^_**2**_G26 modification efficiency and reverses antisuppression in *S*. *pombe*. The results establish a link between RNAP III activity, tRNA production and Trm1 modification activity that impacts tRNA function. We show that this response is conserved, as deletion of *MAF1* from *S*. *cerevisiae* is also accompanied by m^**2**^_**2**_G26 hypomodification, and *maf1-*antisuppression is reversed by over expression of *TRM1*. Finally, we show that human cellular tRNA m^**2**^_**2**_G26 modification efficiency increases with serum starvation or rapamycin treatment and decreases following serum stimulation.

## Results

### Rapamycin decreases tRNA synthesis in a *maf1* ^*+*^-dependent manner in *S*. *pombe*

**Fig 1:**
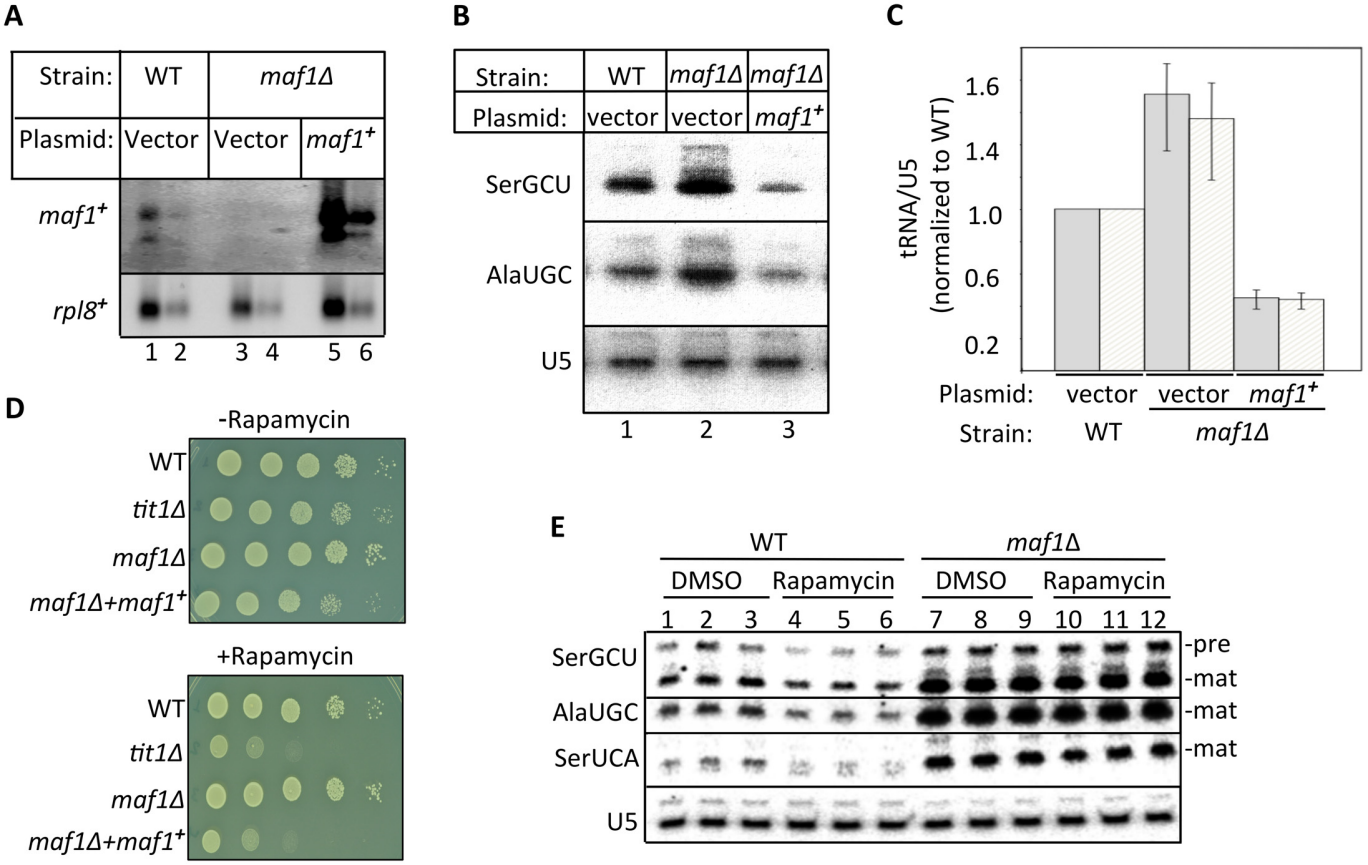
Maf1 is a rapamycin-sensitive regulator of RNAP III-mediated tRNA transcription in *S*. *pombe*. A) Northern blot probed for *maf1* ^*+*^ mRNA in parent wild type (WT) strain, *maf1Δ* strain and *maf1Δ* in which *maf1* ^*+*^ is over expressed from a multicopy plasmid. The *rpl8* ^*+*^ mRNA serves as a loading control. Each sample was loaded in duplicate at 2X and 1X. B) Northern blot analysis of tRNASerGCU, tRNAAlaUGC and U5 snRNA loading control on the same blot from the three strains indicated above the lanes. C) Quantitation of the tRNAAlaUGC (white bar) and tRNASerGCU (grey) transcripts from three northern blots, including from panel B, using U5 snRNA on the same blots for calibration. Error bars indicate standard deviations of three experiments. D) Spot assay showing growth of *S*. *pombe* strains in minimal media (EMM) with or without rapamycin at 100 ng/ml. E) Northern blot comparing the tRNA transcripts indicated in WT and *maf1Δ* cells one hour after the addition of rapamycin or DMSO carrier alone to the liquid culture media.

Unlike for all other species examined, *S*. *pombe* is naturally resistant to the growth inhibitory effect of rapamycin [26]. Thus, it was important to determine if *maf1* ^*+*^ regulates tRNA production in *S*. *pombe* and if it does so under TOR control. We created a *maf1-*deletion strain and showed that it lacked *maf1* ^***+***^ mRNA relative to wild type (WT; Fig 1A, WT/vector vs. *maf1Δ*/vector). Ectopic over-expression of plasmid-borne *maf1* ^***+***^ in *maf1Δ* increased *maf1* ^***+***^ mRNA about 4-fold relative to endogenous *maf1* ^***+***^ in WT cells (Fig 1A). Levels of tRNAAlaUGC and tRNASerGCU were increased in *maf1Δ* relative to WT but decreased relative to WT when *maf1* ^***+***^ was over expressed (Fig 1B). Quantitation of these tRNAs relative to U5 snRNA, a RNAP II transcript on the same blot, from triplicate cultures on triplicate blots revealed that three levels of *maf1* ^***+***^ expression led to three levels of tRNA expression (Fig 1C).

We analyzed the effect of the TOR inhibitor, rapamycin on *maf1Δ* cell growth. Deletion of *tit1* ^***+***^, encoding the tRNA isopentenyltransferase (*MOD5* homolog, see below), which forms i6A37 on some tRNAs, is known to cause sensitivity to rapamycin [14] and served as a control (Fig 1D). In media lacking rapamycin, these strains exhibited relatively similar growth (Fig 1D, upper panel). While the control *tit1Δ* was sensitive to rapamycin, *maf1Δ* was insensitive (Fig 1D, lower panel). By contrast to *maf1Δ*, the cells over-expressing *maf1* ^***+***^ were sensitive to rapamycin (Fig 1D, lower).

To test whether *S*. *pombe maf1* ^***+***^ regulates RNAP III in response to rapamycin, we analyzed tRNA from triplicate cultures of cells in logarithmic growth to which rapamycin was added and incubated for an additional hour. The northern blot in Fig 1E compares *maf1Δ* and *maf1* ^***+***^ cells using probes to three tRNAs and control U5 RNA. In *maf1* ^*+*^ cells, rapamycin decreased precursor and mature tRNA species relative to DMSO treatment while U5 was unchanged (Fig 1E, lanes 1–3 vs. 4–6). *maf1Δ* cells showed higher levels of tRNAs relative to *maf1* ^*+*^ (Fig 1E, lanes 1–3 vs. 7–9). Rapamycin did not reduce tRNA levels in *maf1Δ* cells (Fig 1E, lanes 7–9 vs. 10–12). This established that the ability of *S*. *pombe* to repress tRNA production in response to rapamycin is dependent on *maf1* ^*+*^, as in other species. In addition, we conclude that *maf1* ^*+*^ over-expression causes slow growth and rapamycin sensitivity, likely due at least in part, to decreased tRNA production.

### Deletion of *S*. *pombe maf1* ^*+*^ causes paradoxical antisuppression

tRNA-mediated suppression (TMS) of adenine synthetic genes prevents accumulation of a red pigmented metabolic intermediate and is useful for studying biogenesis and activity of sup-tRNAs (reviewed in [27]). Because all yeast tRNA genes including sup-tRNAs share similar promoters, and *maf1*-mutants are expected to activate RNAP III globally, *maf1-*antisuppression has been an unexplained paradox [22,23]. *S*. *cerevisiae* Maf1 was noted to affect cellular localization of Mod5 which carries out i6A37 formation, leading to the possibility that sup-tRNA i6A37 hypomodification is responsible for *maf1-*antisuppression [19].

**Fig 2:**
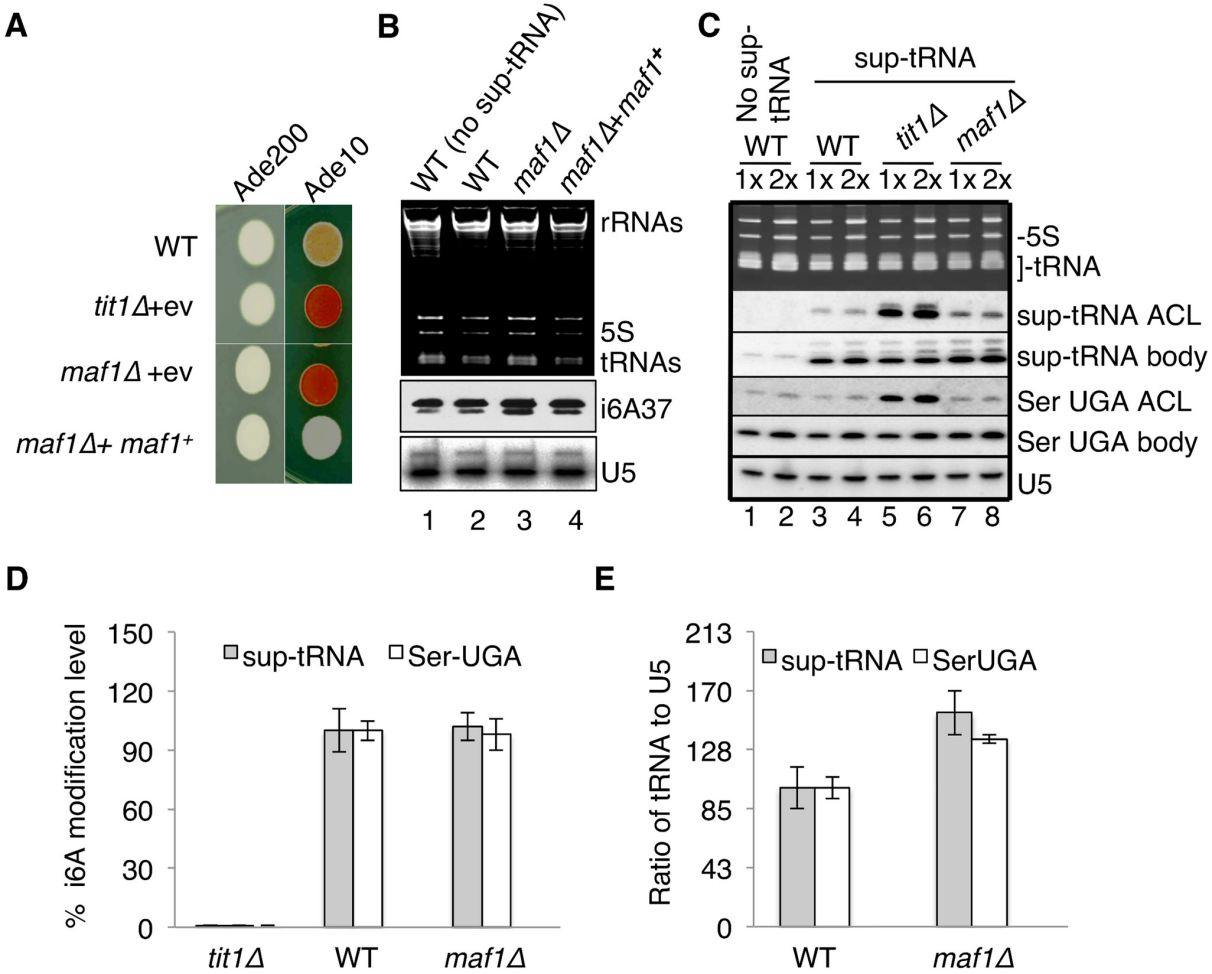
Lack of i6A37 is not responsible for *maf1-*antisuppression phenotype. **A)** tRNA mediated suppression (TMS) in WT (wild-type, i.e., *maf1* ^***+***^), *tit1Δ* (lacking the tRNA A37-isopentenyltransferase-1 gene, *tit1* ^***+***^, see text) and *maf1Δ* cells in excess or limiting adenine (Ade200 vs. Ade10; 200 vs. 10 mg/L, respectively); transformed with empty vector (+ev) or expression vector for *maf1* ^***+***^. **B)** Midwestern blotting of RNA from *maf1* ^***+***^ and *maf1Δ* cells using anti-i6A antibody, and subsequent probing for U5 snRNA as loading control. **C)** Monitoring *in vivo* i6A37 level by PHA6 (positive hybridization in the absence of i6A modification, see text) assay in sup-tRNASerUCA (sup-tRNA) and other RNA as indicated. 1X, 2X = 5, 10 ug total RNA. **D)** Graphic plot of quantification efficiencies in the three *S*. *pombe* strains: % modification = [1− (ACL*tit1* ^***+***^/BP*tit1* ^***+***^)/(ACL*tit1Δ/*BP*tit1Δ*)] X 100. ACL, anticodon loop probe; BP, body probe. **E)** Quantification of steady state levels of the sup-tRNASerUCA and tRNASerUGA examined in panel C. **D & E:** Error bars reflect standard deviations for three experiments.

We examined TMS in *S*. *pombe maf1Δ*, *tit1Δ* and wild-type (WT; *tit1* ^***+***^, *maf1* ^***+***^) strains all of which contain the opal suppressor sup-tRNASerUCA and the opal suppressible allele, *ade6-704*. On limiting adenine, *maf1Δ* exhibited antisuppression relative to WT, similar to *tit1Δ* [28] (Fig 2A, Ade10). Significantly, over-expression of *maf1* ^***+***^ in *maf1Δ* not only reversed antisuppression, it caused more suppression than in WT (Fig 2A, Ade10). Thus as RNAP III activity decreases from high to intermediate to low, sup-tRNASerUCA-mediated TMS activity goes in the opposite direction, from low to intermediate to high.

### i6A37 modification of sup-tRNASerUCA is intact in *maf1Δ* cells

As noted, i6A37 hypomodification was suggested to cause *maf1*-antisuppression [29,30]. Availability of methods to monitor i6A37 on total tRNAs and on specific tRNAs allowed us to test whether *maf1* ^***+***^ deletion led to decreased i6A37. Three tRNAsSer that decode serine UCN codons comprise the major i6A37-tRNA component in *S*. *pombe* while the shorter length tRNATyr and tRNATrp comprise a minor component [28]. Midwestern blotting using anti-i6A antibody [31] showed that the i6A37 content of tRNA differed only slightly among the strains (Fig 2B). No signal was observed with this antibody in *tit1Δ* as expected [28]. The blot was also hybridized with an oligo-DNA probe complementary to U5 RNA as a control (lower panel).

To examine i6A37 levels in sup-tRNASerUCA, we used a PHA6 assay (positive hybridization in the absence of i6A37 modification) in which the northern blot signal intensity increases as i6A37 content decreases because the isopentenyl modification interferes with annealing of a probe targeted to the ACL (anticodon loop), while a 'body' probe to the pseudo-U stem loop of the same tRNA serves as an internal control [14,28,32]. The EtBr stained gel in the upper panel of Fig 2C was blotted, probed, stripped and rehybridized with probes to the RNA species indicated to the right of the other panels. Specificities of the sup-tRNASerUCA ACL and body probes were revealed by paucity of signal in lanes 1 and 2 representing a strain that lacks the sup-tRNASerUCA allele. The ACL probe detected relatively high levels of sup-tRNASerUCA in *tit1Δ* as expected, reflecting lack of the i6A37 modification, but relatively low levels in *maf1Δ* and WT reflecting efficient i6A37 modification. A similar pattern of ACL vs. body probe signal was observed in WT, *tit1Δ* and *maf1Δ*, for endogenous tRNASerUGA which also carries i6A37 [28]. Quantification of sup-tRNASerUCA i6A37 modification efficiency for duplicate samples revealed that it was comparable in *maf1Δ* and *maf1* ^*+*^ WT cells (Fig 2D). We also quantified steady state levels of the tRNAs as monitored by their body probes relative to the U5 control, which confirmed that both were elevated in *maf1Δ* relative to *maf1* ^*+*^ cells (Fig 2E).

The cumulative data suggest that deletion of *maf1* ^*+*^ leads to a decrease in the specific activity of sup-tRNASerUCA for TMS while over-expression of *maf1* ^*+*^ increases its specific activity for TMS. However, the decrease in sup-tRNASerUCA activity in the *maf1Δ* strain is not due to i6A37 hypomodification.

### tRNA-HydroSeq reveals precursor and mature tRNAs

Limitations to high throughput sequencing of tRNAs include inefficient adapter ligations due to secondary structure as well as the multiple modifications that cause reverse transcriptase to pause at each, processively diminishing generation of full length sequence reads. Recent advances have overcome some of the limitations by removing a subset of the blocking modifications by pre-treatment with specific demethylases and/or by use of highly processive thermostable reverse transcriptase [33,34]. Another approach used limited alkaline hydrolysis of total RNA and subsequent mining of reads corresponding to tRNAs [35]. Thus, by generating 19–35 nt hydrolysis products followed by adapter ligation, each fragment will have less potential to form secondary structures and significantly fewer modifications for reverse transcriptase to get past (S1 Fig). We introduced a modification to the Karaca et al. method [35]; namely, purification of tRNA prior to hydrolysis. This was followed by adapter ligation to the fragments, reverse transcription, PCR amplification, and sequencing using Illumina HiSeq technology (S1 Fig).

*S*. *pombe* contains 171 tRNA genes that produce 61 unique tRNA sequences comprising 45 anticodon identities (S1 Table). Although multicopy tRNA genes encode identical mature tRNAs they typically differ in the precursor sequences of 5’ leader, 3’ trailer and/or intron if present [36]. By this account, 150 of the 171 tRNA genes are unique. Sequence reads were first mapped to a reference list representing the 61 unique mature tRNA sequences. The remaining reads were then aligned to a reference list representing the 150 unique precursor-tRNA gene sequences. The total read counts listed in S1 Table provides evidence for expression of all of the tRNA genes in *S*. *pombe*.

**Fig 3:**
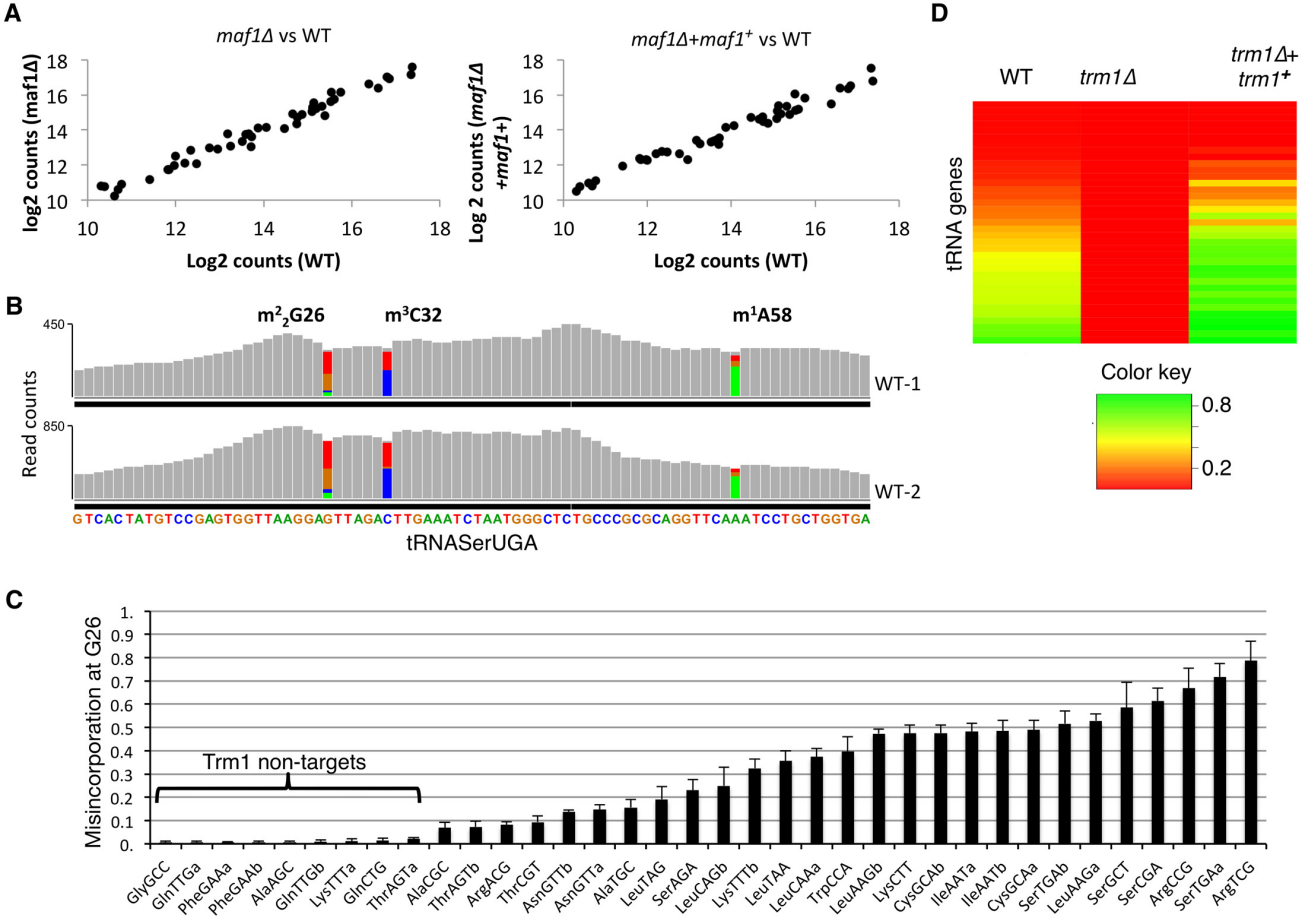
tRNA-HydroSeq identifies some tRNA modifications as misincorporations. **A)** Scatter plots showing normalized counts of *maf1Δ* vs. WT (left), and *maf1Δ+maf1* ***+*** vs. WT (right) cells. **B)** The tRNASerUGA track profiles from IGV display software for two WT strain replicates (WT-1 and WT-2) showing misincorporations as colored bars. IGV introduced color if ≥15% mismatch is detected relative to the reference gene sequence. Color key: green = A, red = T, orange = G, blue = C. Sequence read counts are indicated on the Y-axis. **C**) G26 misincorporations for the 36 *S*. *pombe* tRNAs that have G at position 26, arranged left to right according to misincorporation level. Error bars indicate standard deviation of 4 samples. **D**) Heatmap illustrating misincorporation levels in WT, *trm1Δ*, and *trm1Δ+trm1* ^***+***^ over-expression cells.

Quantitation using DEseq [37] revealed good correlations of the tRNA expression profiles from WT, *maf1Δ* and *maf1Δ*+*maf1* ^*+*^ cells (Fig 3A). This would be expected of Maf1 as a global regulator of RNAP III (Discussion).

### tRNA-HydroSeq identifies some base modifications as misincorporations

tRNA-HydroSeq data included high levels of nucleoside misincorporations at specific positions in specific tRNAs. An example for tRNASerUGA using the IGV display tool is shown in Fig 3B; grey bars indicate match to genomic sequence and colored bars reveal positions at which mismatch was ≥15% as set by IGV. These positions were previously noted to undergo base modifications associated with misincorporation by reverse transcriptase [38]. Modifications of this type disrupt potential for hydrogen bonding and normal base pairing (S2A Fig). We saw no misincorporation at i6A37, as this modification would preserve the potential of adenine for hydrogen bonding (S2B Fig), consistent with prior observations (see [39]). Misincorporations observed at G9, G26, C32 and A58 occurred in tRNAs known to carry m1G9, m22G26, m3C32 and m1A58 in yeast and/or other species as well as at position 34 for the eleven tRNAs with encoded A34, consistent with their deamination to inosine (I) [40,41] (S3 Fig).

G26 is modified by N2,N2-dimethylation (m^**2**^_**2**_G26) in many tRNAs by the Trm1 methyltransferase [42]. Of the 36 tRNAs in *S*. *pombe* that have G26, 27 showed significant misincorporation (≥10%) at G26 (Fig 3C). The extent of G26 misincorporation varied with tRNA identity from 10–80% (Fig 3C). Biochemical studies of *S*. *cerevisiae* Trm1 identified determinants of modification including length of the variable loop [43]. Consistent with those data, we found that G26 tRNAs with variable loops of <5nt (e.g., GlyGCC, GlnTTG, GlnCTG, Fig 3C) showed very low misincorporation. These and data described below suggest that the nine G26 tRNAs with misincorporations of ~1% are not Trm1 targets and reflect background (Fig 3C).

To confirm that G26 misincorporations are due to m^**2**^_**2**_G26 modification, we deleted *trm1* ^*+*^ from its genomic locus and examined misincorporation. The heat map in Fig 3D shows that G26 misincorporations were decreased to ≤1% in *trm1Δ* cells, demonstrating that they are due to m^**2**^_**2**_G26. Notably, expression of *trm1* ^*+*^ from a high copy plasmid in *trm1Δ* cells restored the misincorporations to higher levels than in WT cells (Fig 3D). This suggested that a significant amount of Trm1 substrates are not fully modified in WT cells because Trm1 activity is limiting.

### Hypomodification of m22G26 is responsible for *maf1Δ* antisuppression

**Fig 4:**
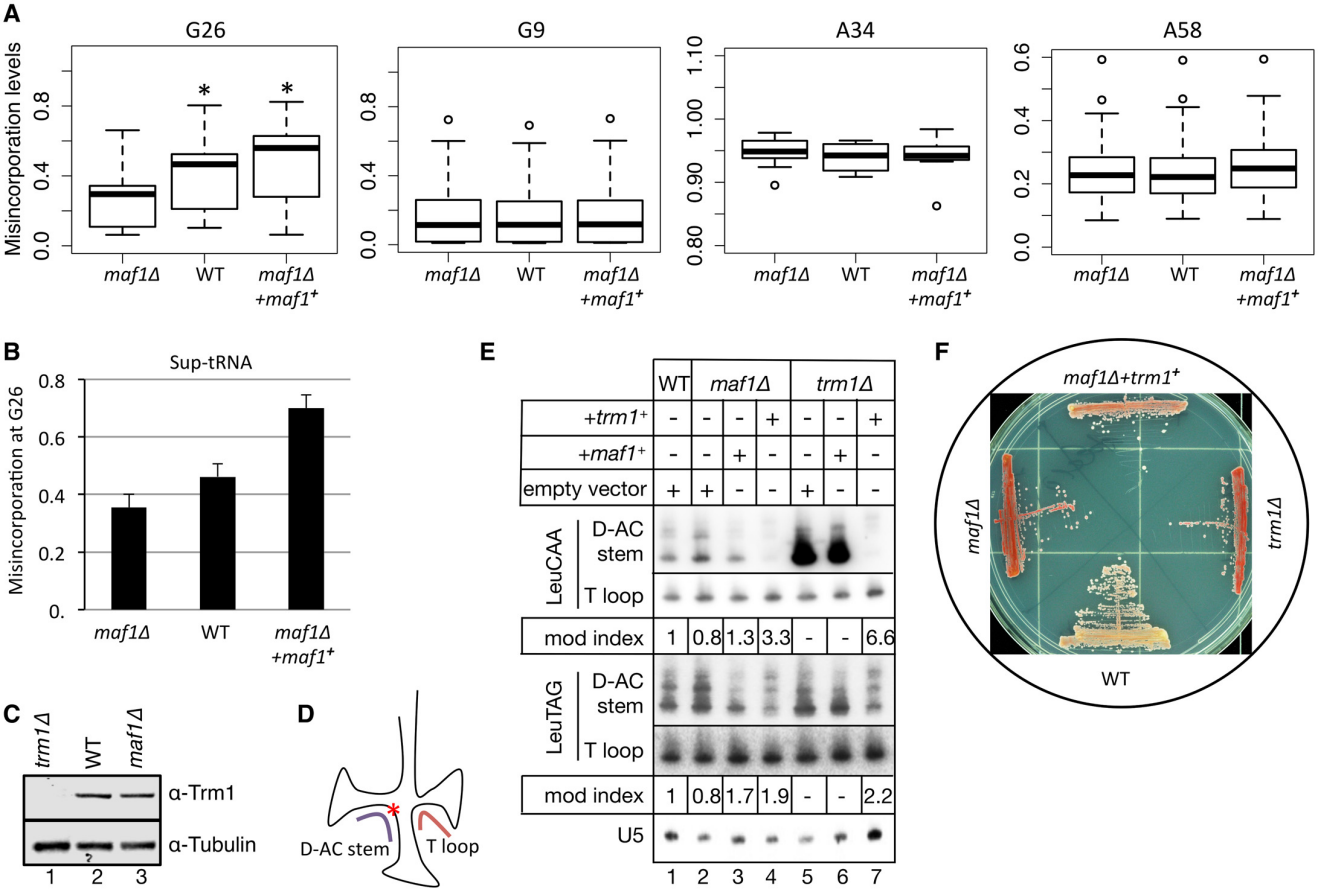
M^2^_2_G26 hypomodification is responsible for the *maf1-*antisuppression paradox. **A)** Box plots showing misincorporations in *maf1Δ*, WT and *maf1Δ+maf1* ^***+***^ strains; G26 box shows misincorporations for 36 tRNAs with G at position 26 (*paired student t test p value <0.001); the G9, A34 and A58 box plots show misincorporations for the tRNA subsets with the corresponding nucleotide identities. **B)** G26 misincorporations for unique reads mapping to sup-tRNASerUCA in *maf1Δ*, WT and *maf1Δ+maf1* ^***+***^ strains. **C)** Western blot analysis of Trm1 levels in the strains indicated above the lanes; tubulin served as a loading control. **D)** Cartoon showing G26 as red asterisk and the two probes used for PHA26 (positive hybridization in the absence of G26 modification) assay. **E**) PHA26 northern blot assay showing sequential probings with oligos to the two different tRNAsLeu indicated to the left; strains are indicated above the lanes and over-expression plasmids are indicated as *+trm1* ^***+***^ *+maf1* ^***+***^ or the control, empty vector. Quantification of T-loop/D-AC stem probe signal is expressed as a modification index where the value of the control, in this case lane 1, set to a value of 1.0, is shown below the lanes of each tRNA panel. **F**) tRNA-mediated suppression (TMS) for WT, *maf1Δ*, *trm1Δ*, and *maf1Δ+trm1* ^***+***^ cells.

Comparative analysis of all tRNAs at all positions in WT, *maf1Δ* and *maf1Δ*+*maf1* ^***+***^ cells revealed that misincorporation levels at G26 were specifically altered in Trm1 target tRNAs in response to three levels of *maf1* ^***+***^ expression in a manner that positively correlated with TMS (Fig 4A, G26 panel, compare with Fig 2A). Misincorporations detected at G9, A34 and A58, reflecting m^**1**^G9, I34 and m^**1**^A58, did not vary with *maf1* ^***+***^ expression (Fig 4A). The specific correlation of G26 misincorporations with three levels of *maf1* ^***+***^ expression and TMS also fits with the finding that Trm1, which appears limiting for G26 modification in WT cells (Fig 3D) may become more so as tRNA levels increase in *maf1Δ* cells, and as shown below, m22G26 is required for suppressor activity.

To more directly link m**22**G26 to Maf1 and its effects on suppression phenotype, we followed three tacks. First, we analyzed sequence reads that uniquely mapped to the sup-tRNASerUCA for G26 misincorporation in WT, *maf1Δ* and *maf1Δ*+*maf1* ^***+***^ cells (Fig 4B). The Sup-tRNASerUCA G26 misincorporations in the three strains reflected the general pattern for the larger subset of Trm1 targets (compare Fig 4A, G26 panel and Fig 4B).

An antibody raised against a C-terminal peptide of *S*. *pombe* Trm1 was used to examine Trm1 levels. This revealed no significant difference in Trm1 levels in *maf1Δ* and WT cells (Fig 4C). The data support a model in which as Trm1 substrates increase with elevated RNAP III activity in *maf1Δ* cells, the efficiency of m**22**G26 modification decreases presumably because a limiting supply of Trm1 cannot keep up with the increase in substrate, and TMS decreases leading to the antisuppression phenotype.

A second tack was to monitor changes in G26 modification levels by an approach other than tRNA-HydroSeq. We devised a northern blot probing method to monitor G26 modification that we refer to as the PHA26 assay (positive hybridization in the absence of G26 modification). Since m**22**G26 modification debilitates normal base pairing [39], we expected it to inhibit annealing of a short probe, designated D-AC stem, to this region of a tRNA while a probe to the variable arm-T loop region of the same tRNA would serve as internal control (Fig 4D). As proof of the PHA26 assay method, the D-AC stem probe showed high signal in *trm1Δ* cells but was decreased upon over-expression of *trm1* ^***+***^ (Fig 4E, tRNALeuCAA, D-AC stem; compare lanes 5 & 7). Although tRNALeuCAA is not as differentially modified in WT, *maf1Δ*, and *maf1Δ*+*maf1* ^***+***^, as is the sup-tRNASerUCA, it does reflect some differential m**22**G26 modification (Fig 4E, D-AC stem vs. T-loop, lanes 1–4). This differential pattern was also seen for tRNALeuTAG (Fig 4E, lanes 1–4); quantification of T-loop/D-AC stem signal is expressed as a modification index, in this case relative to lane 1, below the lanes for each tRNA (Fig 4E). Significantly, over-expression of *trm1* ^***+***^ in *maf1Δ* decreased the D-AC stem probe signal (Fig 4E, compare lanes 2 & 4) demonstrating that Trm1 activity is limiting in *maf1Δ* cells.

PHA26 confirmed that the m**22**G26 content of tRNALeuTAG is lower than for tRNALeuCAA (Fig 4E compare with Fig 3C). Moreover, PHA26 also suggested that tRNALeuTAG is not as ideal a substrate for *trm1* ^***+***^ over-expression as compared to tRNALeuCAA (Fig 4E, compare both tRNAs, lanes 6 & 7, and see below).

A third and most key approach was to determine if Trm1 is limiting for suppression in *maf1Δ* by over-expressing it and assaying for TMS. Deletion of *trm1* ^***+***^ from our WT strain caused antisuppression (Fig 4F). This demonstrated that m**22**G26 modification is required for the suppressor activity of sup-tRNASerUCA, confirming results in *S*. *pombe* with an ochre sup-tRNASerUUA [44]. Most relevantly, over-expression of *trm1* ^***+***^ in *maf1Δ* led to substantial reversal of the antisuppression phenotype in the context of elevated tRNA levels in these cells (Fig 4F). From this we can conclude that m**22**G26 hypomodification due to limiting Trm1 in *maf1Δ* is a determinant of antisuppression in *S*. *pombe*. Thus, changes in RNAP III activity inversely impact m**22**G26 modification and the functional specific activity of sup-tRNASerUCA with consequent phenotype.

### RNAP III mutations that decrease tRNA synthesis also increase m^2^_2_G26 efficiency and tRNA activity

**Fig 5:**
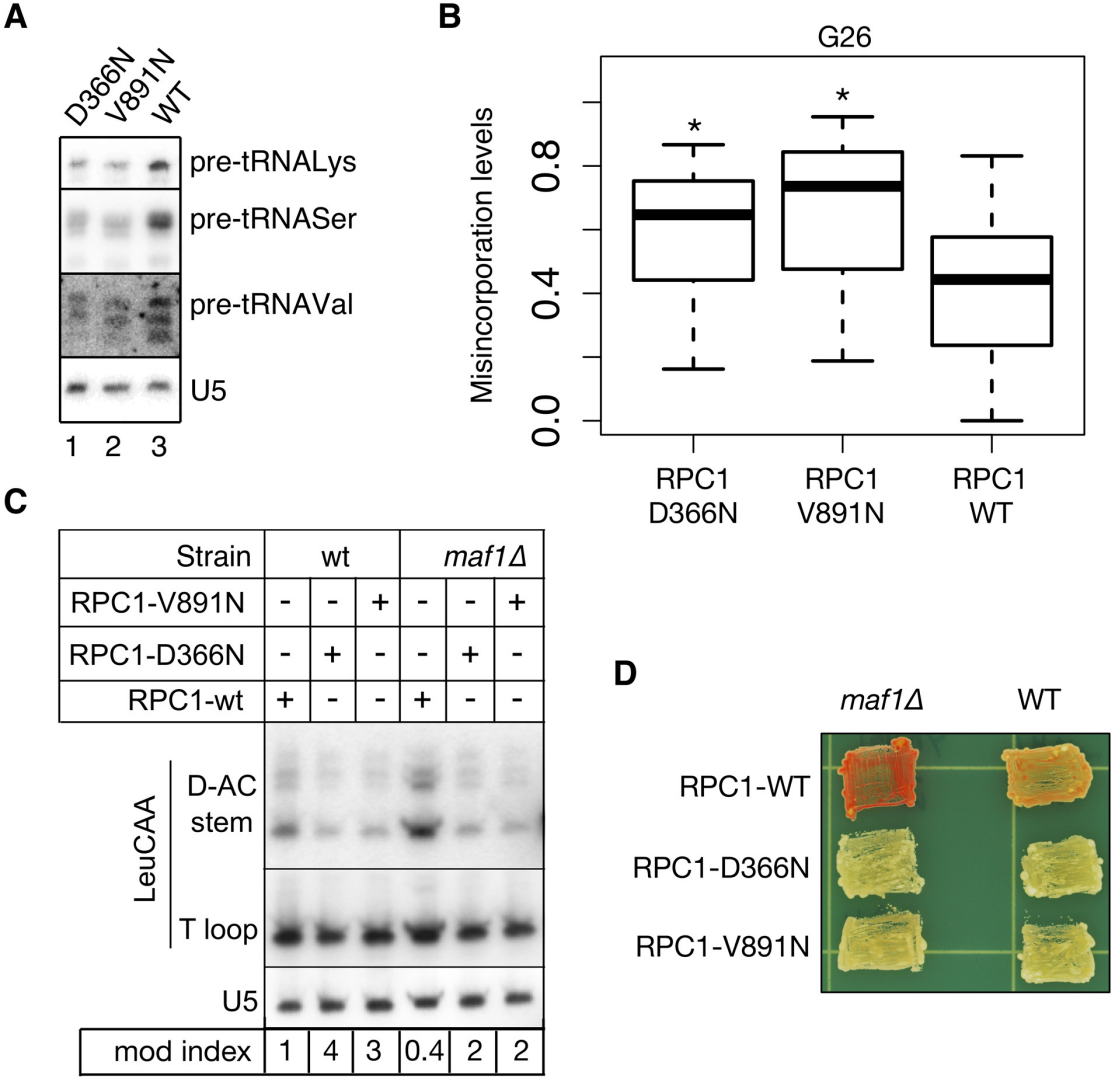
HLD mutations in RNAP III catalytic subunits reduce transcription and increase tRNA m^2^_2_G26 modification efficiency. **A)** Northern blot using probes specific for three precursor-tRNAs indicated to the right of each panel; probe for U5 was used as loading control. **B)** Box plot showing total G26 misincorporation rates for the 27 Trm1 target tRNAs (*paired student t test p value <0.001). **C)** PHA26 northern blot assay for m^**2**^_**2**_G26 modification. **D)** TMS assay for *maf1Δ* and WT cells over expressing the HLD Rpc1 mutations and the Rpc-WT control.

We wanted to ask if a decrease in RNAP III activity by a Maf1-independent mechanism would also affect functional G26 modification. RNAP III is a conserved enzyme whose two largest subunits, Rpc1 and Rpc2, form the catalytic center while other subunits serve supportive and regulatory functions (reviewed in [45]). Certain point mutations in Rpc1 and Rpc2 cause hypomyelinating leukodystrophy (HLD), a tissue-specific developmental disorder, although if they might affect global tRNA transcription has not been reported [46]. Since most of these mutations affect residues invariant from yeast to humans, we introduced them into *S*. *pombe* RNAP III and examined activity after over-expression *in vivo*. *S*. *pombe* RNAP III had previously been used to characterize molecular defects of a zebra fish *rpc2/polr3b-mutant*, *slimjim* that exhibits a tissue-specific phenotype [15,16]. We examined two HLD mutations in Rpc1, D366N in the catalytic center, and V891N at a critical interface with the Rpb5 subunit [45], along with unmutated WT Rpc1, in *S*. *pombe*. We assessed their effects on nascent precursor-tRNA levels, which are widely used to compare RNAP III transcription rates (reviewed in [47]). By this measure, Fig 5A showed for the three tRNA genes examined that the mutations reduced RNAP III transcription relative to WT Rpc1.

According to the model proposed here, as RNAP III activity decreases, reduced amounts of pre-tRNA substrates would better meet the limited supply of Trm1, and their modification efficiency, i.e., the mole fraction of a mature tRNA bearing m^2^_2_G26, would increase. In agreement with this, tRNA-HydroSeq shows significantly more G26 misincorporation in both Rpc1 mutants relative to WT (Fig 5B). This was confirmed by the PHA26 assay, which showed lower D-AC stem probe signal in the Rpc1 mutants relative to Rpc1-WT (Fig 5C, lanes 1–3). The Rpc1 mutations also led to m^**2**^_**2**_G26 hypermodification in *maf1Δ* (Fig 5C, lanes 4–6). Moreover, both Rpc1 mutants robustly reversed antisuppression in the *maf1Δ* strain relative to wild-type Rpc1 and also increased TMS in WT cells (Fig 5D). These data strengthen the model.

### Efficiency of m^2^_2_G26 modification responds to growth/nutrient conditions

**Fig 6:**
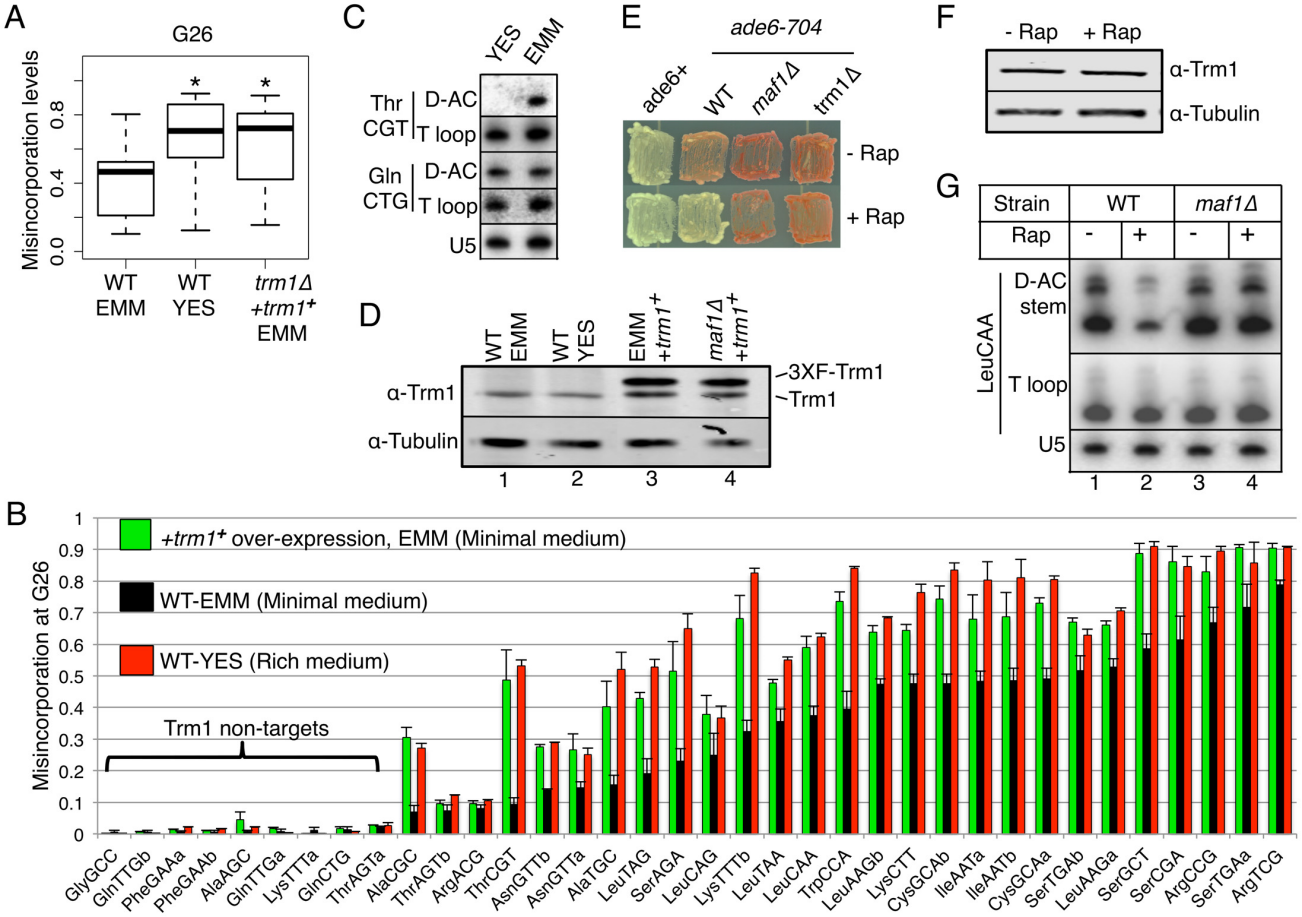
M^2^_2_G26 is regulated by nutrient/growth conditions. **A)** Box plot showing G26 misincorporation levels in WT cells grown in minimal (EMM), rich (YES) media, and *trm1* ^***+***^ over-expression (*trm1Δ*+*trm1* ^***+***^) in EMM as indicated (*paired student t test p value <0.001 relative to WT-EMM). **B)** Bar graph showing G26 misincorporation levels in WT cells in minimal (EMM) and rich (YES) media and +*trm1* ^***+***^ over-expression in EMM. **C)** PHA26 assay for tRNAThrCGT in minimal (EMM) and rich (YES) media. **D)** Western blot analysis for Trm1 in WT cells in minimal (EMM) and rich (YES) media (lanes 1, 2), and +*trm1* ^***+***^ over-expression in EMM (lane 3), as well as *maf11Δ*+*trm1* ^***+***^ in EMM (lane 4); tubulin serves as a loading control. **E)** TMS assay for various strains in +/- rapamycin as indicated to the right. **F)** Western blot analysis for Trm1 in +/- rapamycin as indicated above the lanes; tubulin serves as a loading control. **G)** PHA26 assay on various strains in +/- rapamycin as indicated above the lanes.

As noted above, rapamycin induces nutrient-related stress through the TOR pathway. We examined effects of differing nutrient on m^**2**^_**2**_G26 modification and TMS. tRNA-HydroSeq was performed on wild-type (WT) *S*. *pombe* cells grown in minimal (EMM) and rich (YES) media, the latter known to support faster growth, and in EMM in which *trm1* ^***+***^ was over-expressed. Total G26 misincorporations in Trm1 targets were higher in YES relative to EMM, comparable to cells over-expressing *trm1* ^***+***^ in EMM (Fig 6A).

Examination of individual tRNA G26 misincorporations in minimal (EMM) and rich (YES) media yielded intriguing results. Although some of the greatest increases were in tRNAs that were largely hypomodified in EMM, the response was nonuniform (Fig 6B). Most strikingly was that a very similar pattern of increased misincorporation was observed for cells in YES and when *trm1* ^***+***^ was over-expressed in EMM (Fig 6B). This nonuniform response would appear to reflect individualized substrate-specific responses to an increase in Trm1 activity (Discussion). The nine G26 tRNAs that showed no misincorporation in EMM (≤0.05) also showed no significant increases in misincorporation in YES or with *trm1* ^***+***^ over-expression, providing more evidence that these are not substrates for m^**2**^_**2**_G26 modification, non-targets of Trm1 (Fig 6B).

The increases in G26 misincorporation in WT cells grown in rich (YES) relative to minimal (EMM) media were greater for some tRNAs than others, and this was especially so for tRNAThrCGT (Fig 6B). The PHA26 assay was used to validate this by comparing tRNAThrCGT m^**2**^_**2**_G26 content in YES and EMM. Fig 6C showed that the D-AC stem probe signal for tRNAThrCGT was much lower in YES vs. EMM, while the T-loop probe yielded similar signals, indicating a relatively high level of m^**2**^_**2**_G26 modification in the rich (YES) media. By contrast, tRNAGlnCTG in YES and EMM showed near equal reactivity with its D-AC stem and T loop probes (Fig 6C).

**Fig 7:**
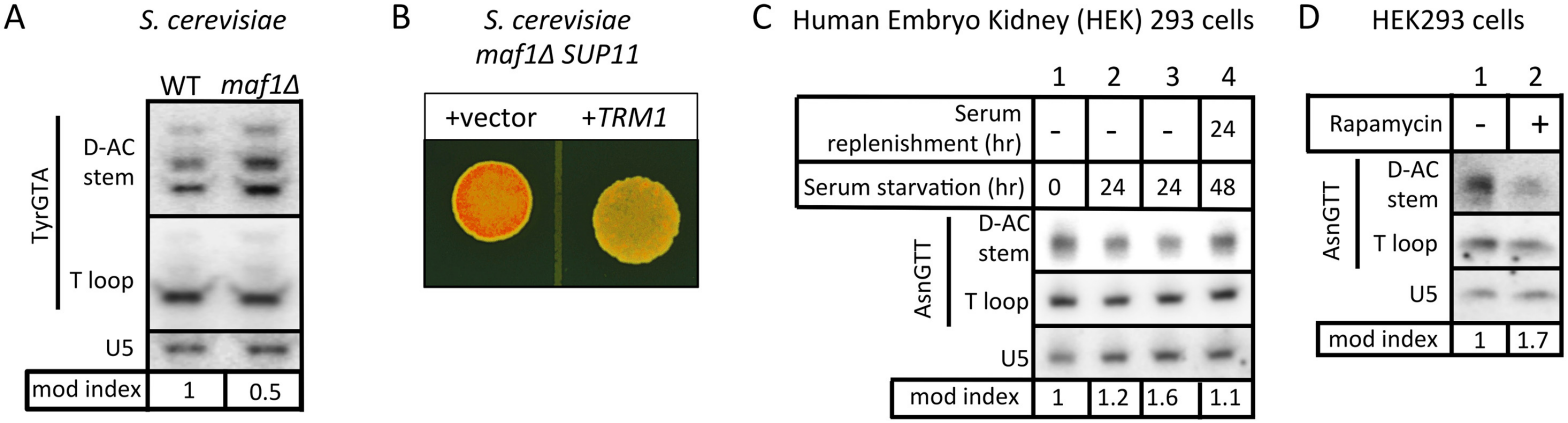
The m^2^_2_G26 modification efficiency response is conserved in *S*. *cerevisiae* and human cells. **A)** PHA26 assay of *S*. *cerevisiae maf1Δ* and WT (*MAF1*) cells. **B)** TMS assay shows that over-expression of *TRM1* reverses antisuppression phenotype of *S*. *cerevisiae maf1Δ* cells. **C)** PHA26 assay of human embryonic kidney (HEK) 293 cells grown for a period of serum starvation or after serum replenishment as indicated above the lanes. **D)** PHA26 assay of HEK293 cells in the presence or absence of rapamycin. Quantitative modification indices are shown for panels A, C and D, described as for Fig 4D.

To gain insight into a potential mechanism controlling the differential m^**2**^_**2**_G26 modification levels in minimal and rich media, we examined Trm1 levels in extracts from the WT cells grown in YES and EMM and the *trm1* ^***+***^ over-expressing cells in EMM by western blotting using tubulin on the same blot as a loading control (Fig 6D). Surprisingly, this showed similar levels of endogenous Trm1 in extracts from cells in grown in YES and EMM (Fig 7D, lanes 1, 2). The over-expressed 3X-FLAG-tagged Trm1 was observed as a slower migrating band in lanes 3 and 4 indicated to the right of Fig 6D. Quantification using Odyssey infrared imaging revealed that the 3X-FLAG-Trm1 accumulated to about 4-fold higher than endogenous Trm1 in the same cell extracts (lanes 3, 4). We conclude that while m^**2**^_**2**_G26 modification efficiency differs dramatically in EMM and YES this is not reflected by Trm1 polypeptide levels detectable by western blotting, whereas over-expression of Trm1 is readily observed.

### Rapamycin modulates tRNA m^2^_2_G26 modification efficiency

We next reasoned that rapamycin, which induces a nutrient-related stress response that includes RNAP III repression [48], would lead to increases in m^**2**^_**2**_G26 modification efficiency and TMS. As alluded to above, the yYH1 (WT; *maf1* ^***+***^ *trm1* ^***+***^) strain is partially suppressed relative to a wild-type *ade6* ^***+***^ allele strain and can therefore reveal an increase or decrease in TMS ([27] and refs therein). Fig 6E shows that while our WT strain is less suppressed relative to *ade6* ^***+***^ but more suppressed than *maf1Δ* in the absence of rapamycin, its suppression increases relative to *maf1Δ* in the presence of rapamycin. Thus, rapamycin led to an increase in sup-tRNASerUCA-mediated suppression in *maf1* ^***+***^ but not in *maf1Δ* or *trm1Δ* cells. Western blotting showed that Trm1 levels were comparable in the rapamycin and control (DMSO) treated WT (*maf1* ^***+***^) cells (Fig 6F). The PHA26 assay showed a lower ratio of D-AC stem to T-Loop signal for tRNALeuCAA in WT (*maf1* ^***+***^) cells treated with rapamycin relative to no rapamycin whereas no difference in D-AC stem signal was observed for the *maf1Δ* cells (Fig 6G). This reflected significant increase in m^**2**^_**2**_G26 modification efficiency specific to rapamycin treated WT (*maf1* ^***+***^) cells. As expected, *maf1Δ* cells showed no response to rapamycin in the assay for sup-tRNASerUCA activity, TMS (Fig 6E), or in m^**2**^_**2**_G26 modification efficiency (Fig 6G).

### The m^2^_2_G26 modification response is conserved

*S*. *cerevisiae MAF1* WT and *maf1Δ* cells were compared for tRNATyr m^**2**^_**2**_G26 content by PHA26 (Fig 7A). The quantitative modification index revealed 2-fold hypomodification in *maf1Δ* relative to WT (*MAF1*) (Fig 7A, mod index under lanes). Ectopic expression of *TRM1* in *S*. *cerevisiae maf1Δ* cells carrying the *SUP11* ochre suppressor-tRNATyrUUA and the ochre-suppressible *ade2-1* allele led to significant reversal of the *maf1-*antisuppression phenotype (Fig 7B).

We also examined m^**2**^_**2**_G26 modification efficiency in human embryonic kidney (HEK) 293 cell tRNAs in response to serum starvation (Fig 7C, lanes 1–3) and treatment with rapamycin (Fig 7D), both of which repress RNAP III and cellular proliferation [49]. In both conditions the m^**2**^_**2**_G26 content of tRNAAsnGTT increased as reflected by the modification index (Fig 7C lanes 1–3, D).

For the experiment in Fig 7C, after serum was added back to the serum-starved cells, their tRNAAsnGTT became less modified (Fig 7C, compare mod index, lanes 3 & 4). These results collectively indicate that the relationship between RNAP III activity and tRNA m^**2**^_**2**_G26 modification efficiency has been conserved through evolution.

## Discussion

This work demonstrates that increases or decreases in global RNAP III activity lead to inverse changes in the efficiency of m^**2**^_**2**_G26 modification of specific tRNAs and this impacts the functional activity of sup-tRNASerUCA in the expression of *ade6-704* mRNA encoding an adenine synthetic enzyme, that produces a suppression phenotype. The collective results show that the link connecting RNAP III and m^**2**^_**2**_G26 efficiency is due to a limiting amount of Trm1, the tRNA G26 dimethyltransferase. The data indicate that this link has been conserved through evolution, among two very highly diverged yeasts and human cells [50].

Maf1 is the conserved central regulator of RNAP III and is under the control of TOR which acts to coordinate growth and proliferation in response to multiple environmental cues including nutrient availability. Repression of RNAP III during starvation is fitting since the energy cost of excessive tRNA synthesis that occurs in the absence of Maf1 is wasted. Indeed, recent analyses indicate that Maf1 is a major mediator of metabolic efficiency [21]. We speculate that the increased efficiency of m^**2**^_**2**_G26 modification that accompanies decreased RNAP III activity may be an economical way to enhance tRNA function under these conditions.

By quantifying tRNA-HydroSeq read counts representing mature tRNAs we found good correlations among the tRNA profiles in WT and *maf1Δ* cells (Fig 3A). An earlier study of *MAF1* in *S*. *cerevisiae* that used microarrays containing tRNA gene sequences found that transcripts encoded by intron-containing tRNA genes were generally much more elevated in the *maf1-*mutant than were tRNAs from intron-less genes [51]. It was later found that the apparent accumulation of intron-containing precursor-tRNAs appeared to result from nonuniform posttranscriptional events such as precursor-tRNA intron processing due to saturation of the tRNA nuclear exportin, Los1 [52]. By using tRNA-HydroSeq to analyze mature tRNAs, good correlations were found among different tRNAs in the WT and *maf1Δ* cells (Fig 3A).

### Trm1 modifies tRNAs in a substrate-specific hierarchical manner

It is interesting that *S*. *pombe* cells in rich media harbor enough Trm1 activity for high efficiency m^**2**^_**2**_G26 modification. However, although m^**2**^_**2**_G26 modification efficiency was significantly higher in rich relative to minimal media (Fig 6B), the levels of Trm1 protein in rich and minimal media were similar (Fig 6D, lanes 1, 2), suggesting that Trm1 activity may be stimulated during growth in rich media by a posttranslational mechanism.

All tRNAs share features that allow recognition by RNase P, RNase Z, and certain other enzymes, but each also harbors features that must contribute to their unique identity. The wide range of m^**2**^_**2**_G26 modification efficiency seen in Figs 3C and 6B is consistent with a hierarchical substrate preference of Trm1. Analysis in EMM and YES indicate increased m^**2**^_**2**_G26 modification efficiency in the latter, more strikingly for some tRNAs than others, and this pattern was mimicked by over-expression of Trm1. These data would appear to reflect a relationship between Trm1 and the distinctive specificities of its many substrates, consistent with biochemical studies [43], but illustrated here for a wide range of unique cellular tRNAs on a tRNAomics-wide scale.

### *N2*,*N2*-dimethyl G26 (m^2^_2_G26) can enhance tRNA function

*S*. *cerevisiae TRM1* exhibits genetic interactions with a number of genes including ones encoding factors involved in tRNA biogenesis and metabolism including several other modification enzymes, the tRNA export factor *LOS1*, the pre-tRNA chaperone and La protein homolog, *LHP1*, and *MAF1* (see this at the *Saccharomyces* genome database, SGD, at http://www.yeastgenome.org/locus/S000002527/interaction).

In the absence of *TRM1* and m^**2**^_**2**_G26, some tRNAs are substrates for surveillance by rapid tRNA decay (RTD) in *S*. *cerevisiae* and the *trm1Δ* cells were shown to exhibit temperature-sensitive growth deficiency [53]. That m^**2**^_**2**_G26 may affect tRNA structure is also consistent with findings that in its absence, tRNALysUUU and tRNATyrGUA become substrates for surveillance by 5'-3' exonuclease Xrn1-mediated RTD [54]. However, although m^**2**^_**2**_G26 modification efficiencies of target tRNAs differed in *S*. *pombe maf1Δ* and *maf1* ^***+***^ cells, their relative steady state levels remained similar (Fig 3A). Moreover, there was no deficiency of sup-tRNASerUCA or tRNASerUGA levels in *maf1* ^***+***^ relative to *maf1Δ* cells (Fig 2C and 2E) despite differences in the percent content of their m^**2**^_**2**_G26. Thus the data indicate that a difference in m^**2**^_**2**_G26 content was a critical determinant to the function of sup-tRNASerUCA in *maf1Δ* and *maf1* ^***+***^ cells. The cumulative results indicate that m^**2**^_**2**_G26 increases the specific activity of the tRNA.

Apparently, m^**2**^_**2**_G26 also increases specific activity of *S*. *cerevisiae* sup-tRNATyrUUA (Fig 7A and 7B), and we believe that it would be reasonable to expect that it may do so for some other tRNAs. We note that the tRNAs whose m^**2**^_**2**_G26 modification efficiencies vary most upon changes in RNAP III activity may impact the translation of some mRNAs more than others, dependent on their cognate codon use bias, and that this may contribute to a phenotype or stress response (see [9,11]) although to attempt to determine if this is decipherable for the subset of m^**2**^_**2**_G26-tRNAs would require substantial bioinformatics and experimental resources and is beyond the scope of this study.

As noted above, evidence that Trm1 acts redundantly with the pre-tRNA chaperone, La protein, suggests that by modifying tRNA with m^**2**^_**2**_G26 it may contribute to proper tRNA folding [24]. G26 resides at the junction between the D-stem and the anticodon stem, and its *N2*-dimethylation, which interferes with normal Watson-Crick base pairing may contribute to prevention of tRNA misfolding. It is also notable that treatment of cells with 5-flurouracil (5FU), which is incorporated into RNA, sensitizes *S*. *cerevisiae* to loss of genes that encode tRNA modification enzymes whose nucleoside targets localize at or near the stems junction, and include *TRM1* [55]. These observations together with evidence that m^**2**^_**2**_G26 can stabilize correctly folded anticodon stems [56], suggest that it may enhance tRNA specific activity by improving fit in the ribosome.

It has been known that inactivation of *MAF1* leads to increased translation fidelity in *S*. *cerevisiae* although the mechanism has been unclear [57]. As a greater percentage of the tRNAs acquire m^**2**^_**2**_G26 in *maf1-*mutants this may be a mechanism that contributes to their increased fidelity. Future studies that compare translational fidelity in *maf1Δ* single mutants with *maf1Δ trm1Δ* double mutants may address this.

*S*. *cerevisiae* Trm1 is tethered to the inner nuclear membrane via a specific amino acid sequence tract [25,58]. A genome-wide global ORF analysis of *S*. *pombe* found Trm1-GFP as nucleoplasmic and mitochondrial with no noted observation of perinuclear localization [59]. Although nuclear residence may limit the time during which a nascent pre-tRNA transcript might have access to acquire the m^**2**^_**2**_G26 modification, retrograde tRNA transport should theoretically allow iterative access to Trm1 (see [54]). Therefore, the mechanism by which cells maintain Trm1 activity in a functionaly limiting amount in minimal media is unclear. In any case, the data suggest that regulation of Trm1 can under certain conditions, differentially impact tRNA activity.

### tRNA-HydroSeq

Others have developed means to identify RNA modifications from deep sequencing data sets [38]. The ablation of G26 misincorporations following deletion of *trm1* ^***+***^ (Fig 3D), provided essential evidence that the correlation of G26 misincorporations with the suspected modification was indeed due to m^**2**^_**2**_G26. Thus, this approach can be used by tRNA-HydroSeq and similar methods together with genetics to obtain quality and quantity information toward studying the biology of certain tRNA modifications. tRNA-HydroSeq detected misincorporations corresponding to m1G9, m^**2**^_**2**_G26, m^**3**^C32, A34I, and m1A58 in tRNAs known to carry these modifications in yeast and/or other species. As was the case for G26, the G9 and A58 misincorporation levels varied in a tRNA-dependent manner (S3 Fig). However, unlike G26, we found no functional correlation of the other modifications with *maf1* ^***+***^ expression, RNAP III activity, and suppression phenotype. Notably, A34I was distinguished from the other misincorporations in that there was uniformly efficient misincorporation in all tRNA substrates (S3 Fig). In addition, we found an intriguing tRNA-specific effect on A34I for the Rpc1 mutants and for growth media (S4 Fig). For 10 of the 11 tRNAs with A34 there was no significant difference in the Rpc1 mutants whereas a single tRNA, SerAGA showed reduced A34 misincorporations in both mutants (S4A Fig). We also observed significant difference in A34 misincorporation unique to tRNASerAGA in YES vs. EMM (S4B Fig). While the basis of this specificity and its significance is unknown, we note that while tRNASerAGA can decode both UCC and UCU codons and that the ratios of these differ greatly in high-expression vs. low-expression mRNAs [60], only the UCC (wobble) codon decoding is dependent on I34. These data provide examples of utilities of tRNA-HydroSeq beyond the ability to follow G26 modification.

### Pathogenic RNAP III mutations

Although mutations in RNAP III catalytic subunits cause HLD, their effects on the RNAP III transcriptome had not been reported [46]. We recreated two of these mutations in *S*. *pombe* at residues highly conserved from yeast to man. The mutations caused decreased transcription of the three tRNA genes examined and were associated with alterations of m^**2**^_**2**_G26 and A34I modification efficiencies.

We wish to emphasize that these HLD mutations were introduced into *S*. *pombe* as a means to globally decrease RNAP III activity for the purposes of this study. Nonetheless it is tempting to speculate that these mutations might have similar effects on human RNAP III activity and possibly analogous changes in tRNAs. The results suggest that while RNAP III activity may be increased or decreased globally due to a number of mechanisms, the output with regard to tRNA activity may be asymmetric or nonumiform. In such cases, nonuniform alterations of tRNA activities on different subsets of codon-biased mRNAs may contribute to phenotype [1,9,10].

### m^2^_2_G26 hypomodification causes *maf1*-antisuppression

To the best of our knowledge *maf1-*antisuppression had been observed only in *S*. *cerevisiae* and for ochre sup-tRNAs of Tyr identity [22,30], which in *S*. *cerevisiae* carry m^**2**^_**2**_G26. The present work extends this to *S*. *pombe* and sup-tRNASerUCA. The *S*. *cerevisiae maf1-1* mutant was isolated from a *mod5-*mutant deficient for cytoplasmic i6A37 modification [29]. However, the hypothesis that i6A37 hypomodification was responsible for *maf1*-antisuppression had not been tested experimentally [19]. Our data show no i6A37 deficiency in *S*. *pombe maf1Δ* cells and that antisuppression occurs despite efficient i6A37 modification of sup-tRNASerUCA. Instead the data show that m^**2**^_**2**_G26 hypomodification of sup-tRNASerUCA is responsible for *maf1*-antisuppression. In *S*. *cerevisiae*, the tRNATyrGUA, from which the ochre suppressor *SUP11* was derived, contains G26 and is hypomodified in *maf1Δ* cells (Fig 7A). In *S*. *pombe*, sup-tRNASerUCA contains G26 and is hypomodified in *maf1Δ* cells. Over-expression of Trm1 in both *S*. *cerevisiae* and *S*. *pombe* substantially reverses their *maf1-*antisuppression phenotypes (Figs 4F and 7B). We note that antisuppression reversal by Trm1 over-expression was incomplete. Among other possibilities this suggests that other factors involved in TMS may be limited for suppression in the context of increased tRNA synthesis in *maf1Δ* cells.

Treatment of *S*. *pombe maf1* ^***+***^ cells with rapamycin, which represses RNAP III via *maf1* ^***+***^, increased suppression accompanied by m^**2**^_**2**_G26 hypermodification. Similarly, human cells treated with rapamycin showed robust increase in m^**2**^_**2**_G26 modification content. Serum-starvation led to increased m^**2**^_**2**_G26 modification that decreased with serum replenishment (Fig 7C). Likewise, *S*. *cerevisiae* tRNATyr was m^**2**^_**2**_G26 hypomodified in *maf1Δ* relative to *MAF1* cells. In *S*. *pombe*, unlike the other modifications examined, only m^**2**^_**2**_G26 varied with *maf1* ^***+***^ expression concordant with TMS activity. This specificity is noteworthy since although a genetic screen uncovered *GCD10* and *TRM10*, responsible for m1A58 and m1G9 tRNA modifications, as well as *TRM1* [61], our data showed that m1A58 and m1G9 were not altered in *maf1Δ* relative to WT or *+maf1* ***+*** whereas m^**2**^_**2**_G26 levels were. In summary, the link between RNAP III activity and m^**2**^_**2**_G26 modification efficiency appears to be specific and conserved.

## Materials and Methods

### Yeast and growth conditions

*S*. *pombe* strains used are listed in S2 Table. Cells were grown in minimal media (EMM lacking uracil) or in rich media (YES) to an OD_600_ of 1.0. *S*. *pombe* cells were seeded to an OD600 of 0.4, and ten-fold dilutions were plated on the appropriate media as indicated. For liquid growth, overnight cultures were diluted to OD600 of 0.25 and incubated for two hours after which rapamycin (AG Scientific Inc., R1018) at 200 ng/ml, or DMSO alone (Sigma, D2650) was added one hour prior to RNA isolation.

### Cloning *S*. *pombe trm1+* for expression

The *trm1* ***+*** open reading frame was amplified from *S*. *pombe* genomic DNA using a forward primer containing sequence for 3X FLAG peptide and XhoI site and a reverse primer with XmaI site. The PCR products were digested with XhoI and XmaI and ligated into XhoI-XmaI digested pREP4X.

### Cloning *S*. *pombe maf1+* for expression

The *maf1+* gene and its 600 bp upstream region was PCR amplified from genomic DNA and cloned into the XhoI-PstI sites of pRep4X (removing the *nmt1+* promoter) resulting in plasmid CB235.

### RNA preparation

Total RNA was isolated using hot phenol. In short, 50 ml cultures grown from an OD600 of 0.1 to 0.5 were harvested, washed with water and resuspended in 300 μl TES buffer (10 mM Tris Cl pH 7, 10 mM EDTA, 1% SDS). 300 μl water-equilibrated phenol was added and incubated at 65°C for 45 minutes, with vortex every 15 min. To the samples, 300 μl chloroform was added and centrifuged. Supernatant was extracted twice with acid-phenol-chloroform and once with chloroform before precipitation with ethanol.

### Northern blot assays

Total RNA was resolved in 6% NuPAGE TBE-Urea gel (Life Technologies) and transferred to positively charged nylon membranes. Probing and washes were done as described previously [14].

### Midwestern blotting

Using affinity-purified anti-i6A from rabbit [31] (a kind gift of Anita Hopper, OSU) at 1:50 and processed for chemiluminescence was as described [28].

### tRNA-HydroSeq

For tRNA isolation, 50 μg of total RNA was separated on a 6% TBE-Urea polyacrylamide gel (21 cm X 18 cm X 0.15 cm) followed gel purification of RNA shorter than 5S rRNA. 300 ng purified tRNA was hydrolyzed in 10 mM bicarbonate buffer pH 9.7 at 90°C for 5 min. The hydrolyzed RNA was dephosphorylated using calf intestinal alkaline phosphatase (NEB) followed by 5' phosphorylation by T4 polynucleotide kinase (NEB). Barcoded, pre-adenylated, 3’ blocked Illumina adapters were ligated to the 3’ end using T4 Rnl2(1–249)K227Q enzyme (NEB). After heat inactivation, all ligated samples were pooled in ethanol and precipitated. In parallel, two size marker RNA oligos of 19 and 35 nt were radiolabeled using T4 polynucleotide kinase and ligated to pre-adenylated adapter and pooled. Ligated tRNA samples and markers were resolved in a 15% TBE urea acrylamide gel followed by gel purification of tRNA samples between 19 nt and 35 nt guided by the marker lanes. 5’ adapter was ligated to the gel purified samples using T4 RNA ligase I (Thermo). RNA with ligated 5’ and 3’ adapters were gel purified, subjected to reverse transcription (Superscript III, Life Technologies), amplified by PCR (10–12 cycles) and the band was gel purified and sequenced using Illumina HiSeq 2500.

### Sequence read alignment and quantitation

tRNA read depths for all samples generally varied with tRNA gene copy number; from 278 for the lowest read from the single copy ArgCCG gene in one replicate to greater than one-million reads for highly abundant tRNAs. Sequence reads were mapped to a reference file comprising sequences of all 61 unique mature *S*. *pombe* tRNAs. Sequences that did not map to this file were then mapped to a file containing all 150 unique *S*. *pombe* precursor-tRNA genes. Read count tables were made using the mapping data and analyzed using DEseq software.

### Quantitation of mismatches in tRNA reads

The fraction of reads that mismatched the reference gene sequence at each position were tabulated. The average values of the fraction of misincorporation among replicates was calculated and plotted (e.g., Fig 3C).

### Western blotting

*S*. *pombe* cells were grown in 10 ml of the noted media to A600 of 0.7–1, washed with water and resuspended in 400 ul of 20% trichloroacetic acid (TCA). Glass beads (0.5 mm) were added and vortexed for 1 min. The beads were separated from the material, washed with 5% TCA and the wash was pooled with the material recovered. This was centrifuged at 6000 RCF for 10 minutes, the supernatant discarded and the pellet washed twice with 1 ml acetone. The pellet was air dried and dissolved in 400 ul of 1X SDS sample buffer containing fresh beta-mercaptoethanol. Aliquots were resolved on a 4–12% Bis-Tris gel (Life technologies) followed by transfer to PVDF membrane. The blot was blocked for 1 hour with 5% non-fat milk in PBS. Polyclonal anti-Trm1 antiserum raised against the C-terminal peptide, GPKSKPGKRTIAEVDSKS, in rabbit (Thermo Fisher Scientific, Waltham, MA; animal #PA9064, day 56 bleed) was used at 1:500 in blocking buffer with 0.1% Tween 20. Anti-Tubulin (Sigma, #T5168) was used at 1:4000. After 1 hr. the blot was washed 4 times with PBS-Tween. Appropriate secondary Abs (LI-COR) of different fluorescent emittances were used at 1:20,000 in 5% milk solution in PBS with 0.2% Tween-20 and 0.01% SDS for 1 hour followed by 4 washes in PBS-T. The washed blot was scanned using LI-COR Odyssey Clx system and the images processed and bands quantified using ImageStudioLite software.

### *TRM1* cloning and over-expression in *S*. *cerevisiae*

The glyceraldehyde phosphate dehydrogenase (GPD) promoter followed by a 3X-HA tag was amplified from the pYM-16 plasmid (PCR-toolbox, EUROSCARF) and cloned into the SacI-NotI sites of pRS426 to generate the pRS426GPD vector. The *S*. *cerevisiae TRM1* gene starting from the ATG start codon to 855 bp downstream of the stop codon was amplified from genomic DNA and digested with NotI and XhoI present in the primers used. The fragment was inserted in the corresponding site of pRS426GPD. The empty vector (pRS426GPD) and the Trm1 clone were used to transform *S*. *cerevisiae maf1Δ* strain, MB159-4D*Δ* [57] which carries *SUP11* sup-tRNATyrUUA, and plated on SC agar lacking uracil with 10 mg/l adenine.

## Supporting Information

S1 Fig Schematics showing general approach of tRNA-HydroSeq method. (TIF)

S2 Fig Ilustrations of Watson:Crick base pairing disruption from loss of potential for hydrogen bonding due to presence of modification (red). The right column indicates base preferences calculated from misincorporation frequencies detected by tRNA-HydroSeq.
(TIF)

S3 Fig Fraction of reads showing misincorporation at G9 (A), A58 (B) and A34 (C), each in the appropriate subsets of target tRNAs in WT cells. (TIF)

S4 Fig A) A34 misincorporations in all 11 target tRNAs in WT, *maf1Δ*, and the two Rpc1 mutants, D366N and V891. Each of the 11 A34 tRNAs are indicated according to the insets; only tRNASerAGA (red) is altered in the Rpc1 mutants. B) A34 misincorporations in the 11 A34 tRNAs in EMM vs YES. tRNA identies are on the X-axis. Error bars indicate standard deviations for A) and B)
(TIF)

S1 Table Read counts from tRNA-HydroSeq for precursor and mature tRNAs from the different strains examined and their replicates as indicated above the columns. The yYH1 -M1, -M11 and WT-C1 refer to the Rpc1 mutants (see text).
(XLSX)

S2 Table Yeast strains used in this study. (DOCX)
