# Supplementary figures and images for: RNA Polymerase III Output Is Functionally Linked to tRNA Dimethyl-G26 Modification
Source: PLoS Genet. 2015 Dec 31;11(12):e1005671. doi: 10.1371/journal.pgen.1005671 (PMC4697793; doi:10.1371/journal.pgen.1005671)

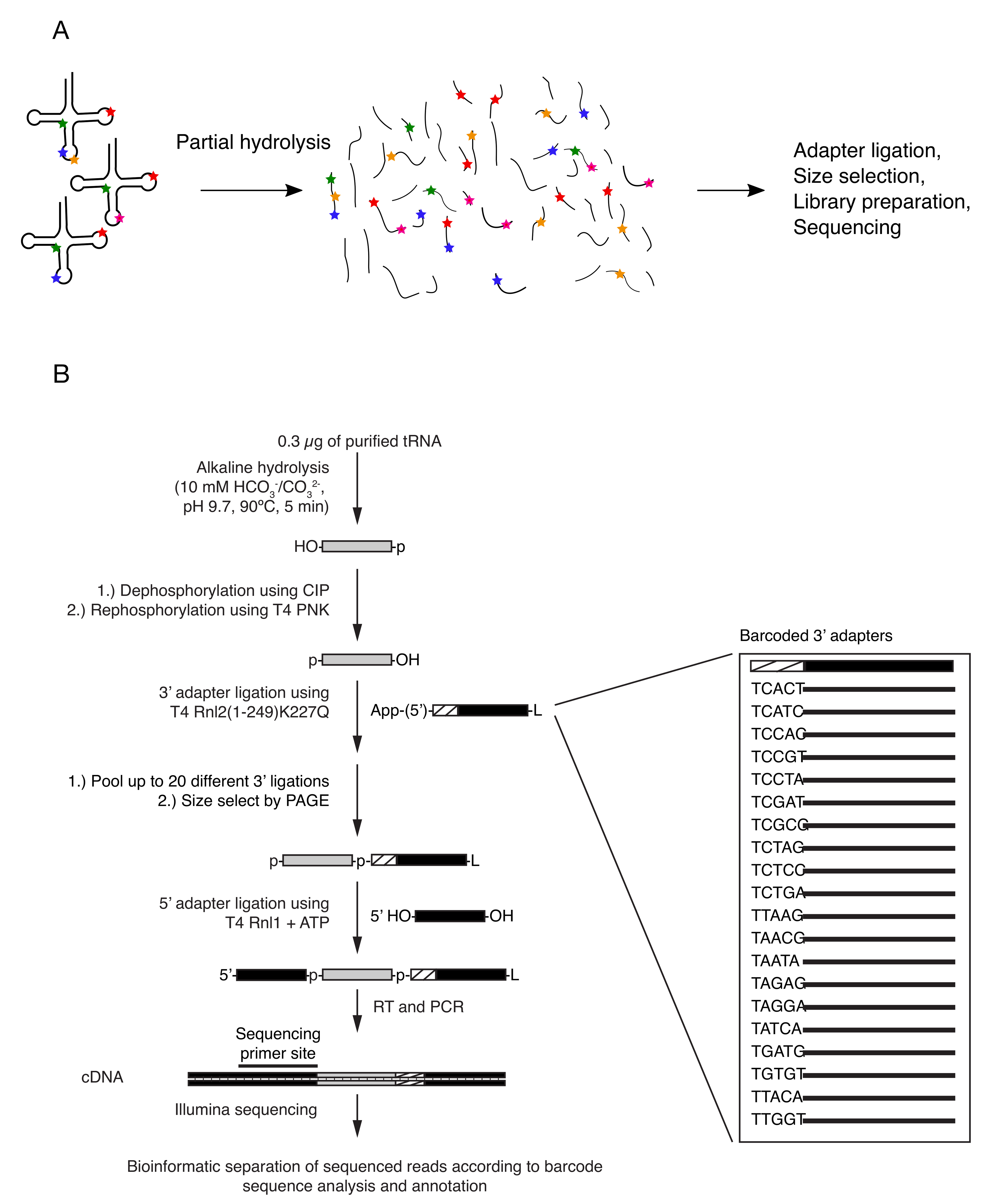

Supplement: S1 Fig — (TIF) [file pgen.1005671.s001.tif]

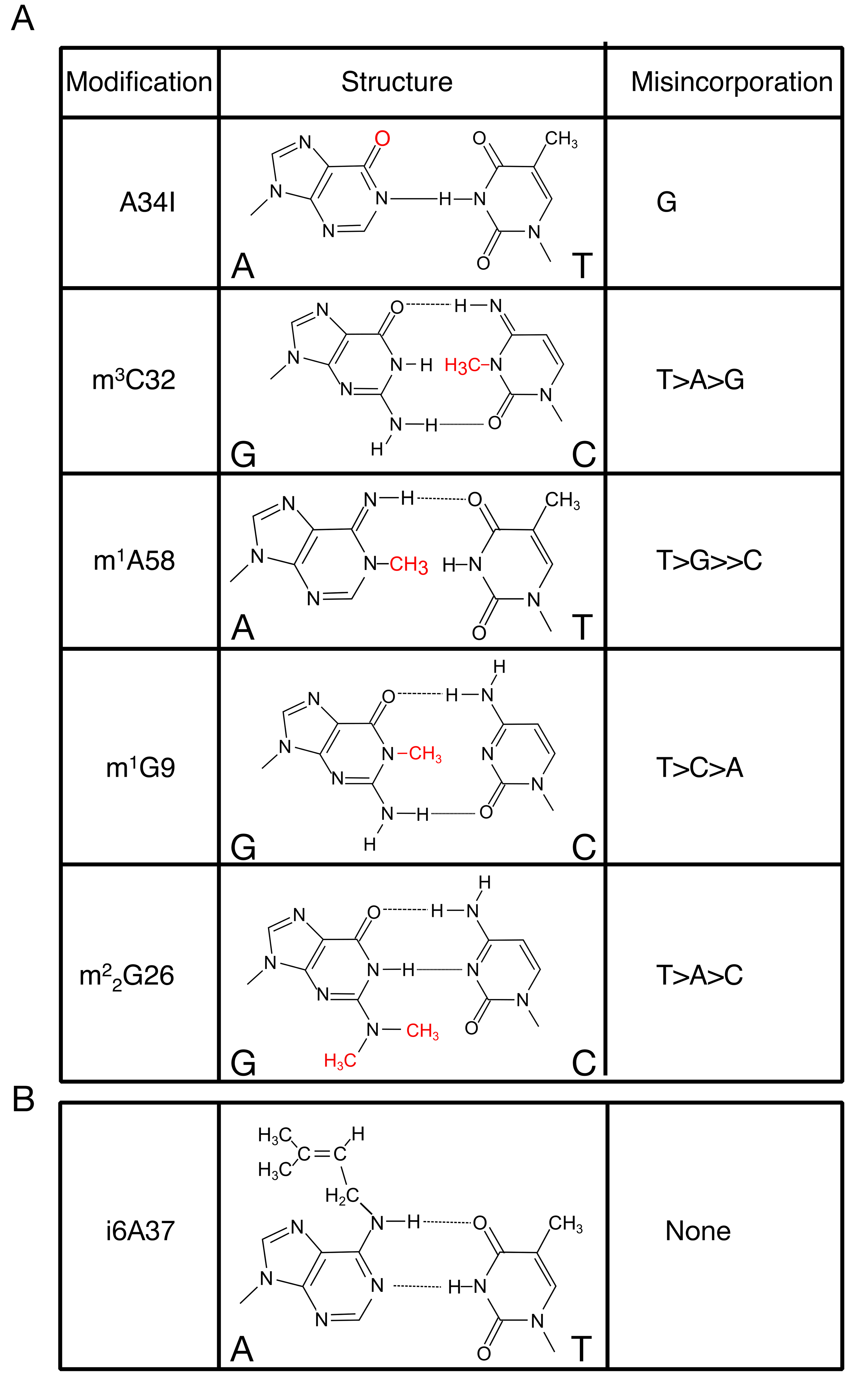

Supplement: S2 Fig — The right column indicates base preferences calculated from misincorporation frequencies detected by tRNA-HydroSeq. (TIF) [file pgen.1005671.s002.tif]

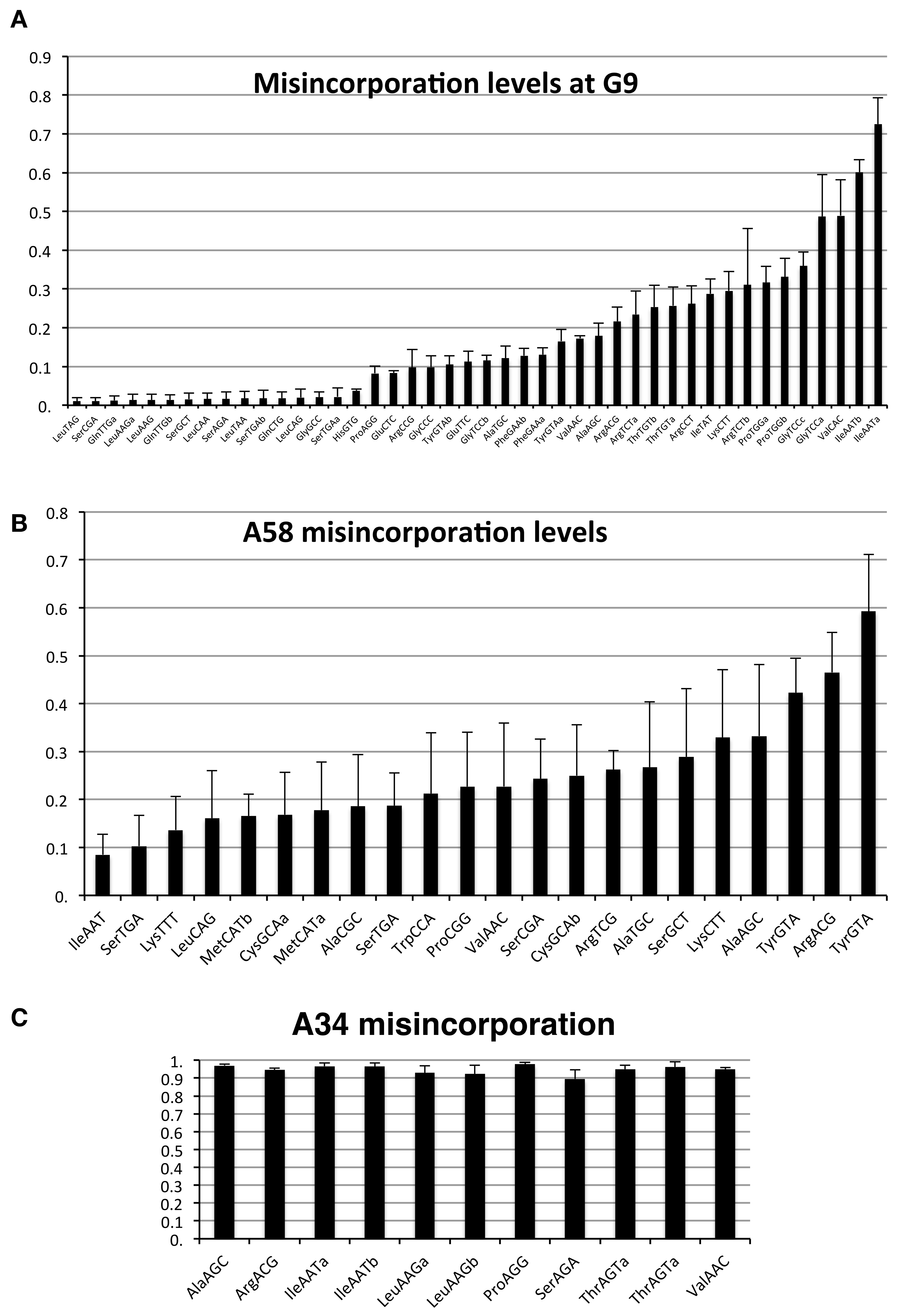

Supplement: S3 Fig — (TIF) [file pgen.1005671.s003.tif]

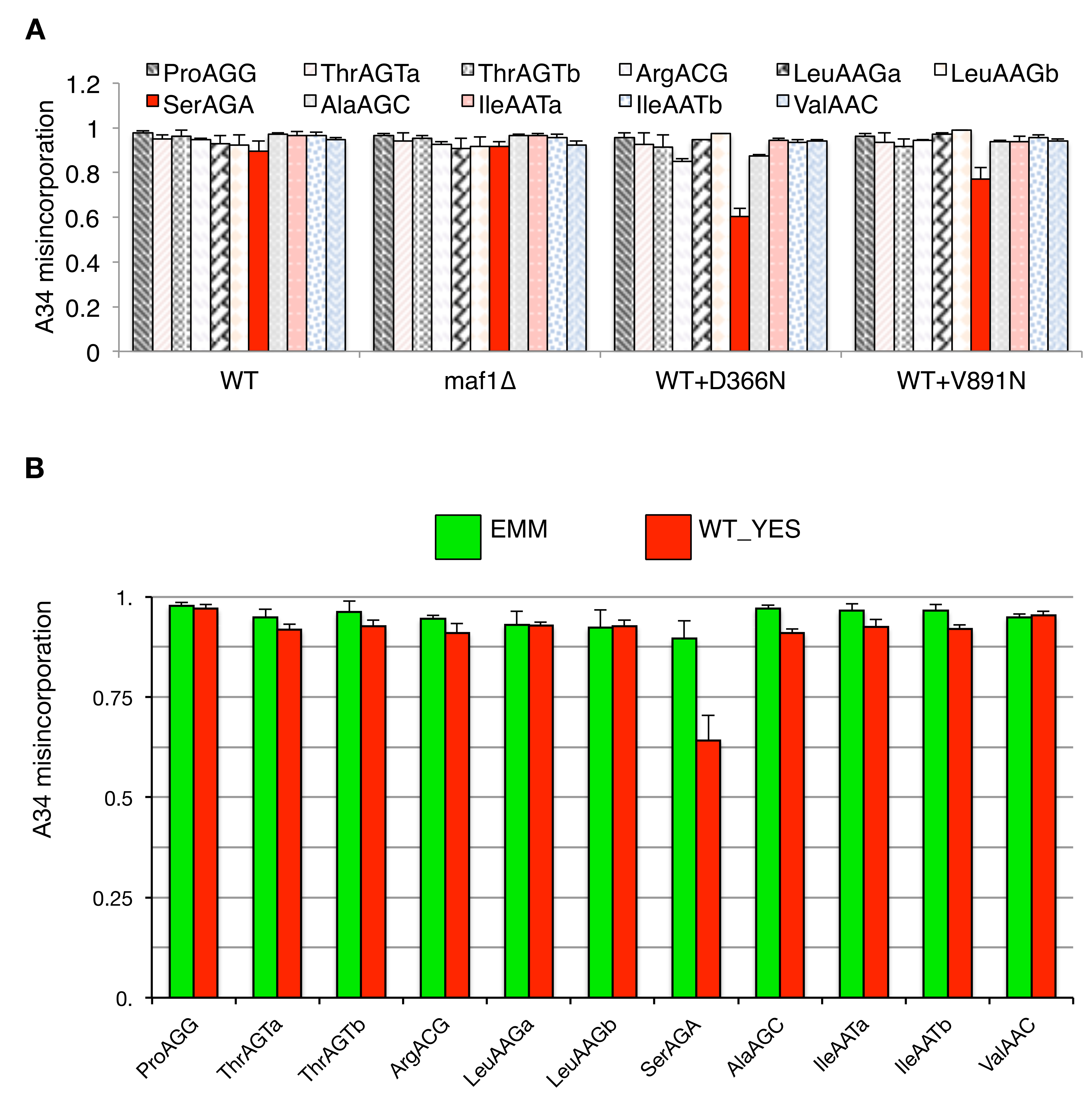

Supplement: S4 Fig — Each of the 11 A34 tRNAs are indicated according to the insets; only tRNASerAGA (red) is altered in the Rpc1 mutants. B) A34 misincorporations in the 11 A34 tRNAs in EMM vs YES. tRNA identies are on the X-axis. Error bars indicate standard deviations for A) and B) (TIF) [file pgen.1005671.s004.tif]
