# Supplementary material for: RNA Polymerase III Output Is Functionally Linked to tRNA Dimethyl-G26 Modification
Source: PLoS Genet. 2015 Dec 31;11(12):e1005671. doi: 10.1371/journal.pgen.1005671 (PMC4697793; doi:10.1371/journal.pgen.1005671)
Supplement: S2 Table — (DOCX) [file pgen.1005671.s006.docx]

| Species | Strain name | genotype |
| --- | --- | --- |
| *S. pombe* | yYH1 (WT) | *h*− *leu1-32*::[*tRNAmSer7T-leu1+*] *ura4-D18 ade6-704* |
| *S. pombe* | yNB1 (*maf1∆*) | *h*− *leu1-32*::[*tRNAmSer7T-leu1+*] *ura4-D18 ade6-704 maf1∆::KanMX6* |
| *S. pombe* | yAG501 (*trm1∆)* | *h*− *leu1-32*::[*tRNAmSer7T-leu1+*] *ura4-D18 ade6-704 trm1∆::KanMX6* |
| *S. pombe* | yAG502 (*ade6+)* | h-, ura4-294, Leu1-32 *ade6+* |
| *S. pombe* | yNB5 (*tit1∆*) | *h*− *leu1-32*::[*tRNAmSer7T-leu1+*] *ura4-D18 ade6-704 tit1∆::KanMX6* |
| *S. cerevisiae* | W3031a (WT) | MATa leu2-3,112 trp1-1 can1-100 ura3-1 ade2-1 his3-11, 15 |
| *S. cerevisiae* | maf1∆ | MATa leu2-3,112 trp1-1 can1-100 ura3-1 ade2-1 his3-11, 15 maf1∆::KanMX6 |
| *S. cerevisiae* | MB159-4DΔ  (maf1∆ SUP11) | MATaSUP11 ade2-1 ura3-1 leu2-3,112 lys2-1 his3 maf1∆::Kan |

Arimbasseri et al., Supplemental Table S2
